# Influence of Nutrient Media Compared to Human Synovial Fluid on the Antibiotic Susceptibility and Biofilm Gene Expression of Coagulase-Negative *Staphylococci* In Vitro

**DOI:** 10.3390/antibiotics10070790

**Published:** 2021-06-29

**Authors:** Stephan Josef Maria Steixner, Christopher Spiegel, Dietmar Dammerer, Alexander Wurm, Michael Nogler, Débora Cristina Coraça-Huber

**Affiliations:** 1 Research Laboratory for Biofilms and Implant Associated Infections (BIOFILM LAB), Experimental Orthopaedics, University Hospital for Orthopaedics and Traumatology, Medical University of Innsbruck, Peter-Mayr-Strasse 4b, Room 204, 6020 Innsbruck, Austria; stephan.steixner@i-med.ac.at (S.J.M.S.); christopher.spiegel@student.i-med.ac.at (C.S.); michael.nogler@i-med.ac.at (M.N.); 2 University Hospital for Orthopaedics and Traumatology, Medical University of Innsbruck, Anichstrasse 35, 6020 Innsbruck, Austria; dietmar.dammerer@i-med.ac.at (D.D.); Alexander.wurm@i-med.ac.at (A.W.)

**Keywords:** biofilm, coagulase-negative *staphylococci*, antibiotic susceptibility, nutrient media, simulated synovial fluid, viability, gene expression, biofilm genes, glucose, diabetes

## Abstract

Bacterial antibiotic resistance and biofilm formation are mechanisms usually involved in the pathogeny of implant-related infections. Worldwide, antibiotic susceptibility tests are usually carried out using nutrient-rich media. Clinical routine laboratories and even research centers use for example EUCAST or CLSI for guidelines. In this study, we investigated the effect of different nutrient media on the antibiotic susceptibility and *ica*ADBC gene expression of bacteria in biofilm. As media, Müller-Hinton Bouillon (MHB), Tryptic Soy Broth (TSB) and human synovial fluid (SF) diluted 1:4 in phosphate buffered saline (PBS), each also supplemented with 1% glucose, were used. The influence of different nutrient media on the antibiotic susceptibility of coagulase-negative *staphylococci* (CoNS) was evaluated by counting of colony-forming units (CFU) and by checking the metabolic activity of the bacteria. We used reverse transcriptase and real-time qPCR to investigate the influence of nutrient media on the biofilm gene expression. We used two-way analysis of variance (ANOVA). *p* < 0.05 was considered to be statistically significant. Significant differences in growth and antibiotic susceptibility were detected in all strains tested among the different media used. The nutrient media showed influence on the cell viability of all bacteria after antibiotic treatment. *Ica*ADBC gene expression was significantly influenced by glucose and all nutrient media. The results highlight the influence of glucose on the antibiotic susceptibility, growth and gene expression of all strains tested. For all strains, a significant difference in bacterial recovery, viability and gene expression were found when compared to biofilm grown in SF.

## 1. Introduction

Due to the rising number of total knee arthroplasties (TKA) and total hip arthroplasties (THA) the rates of periprosthetic joint infection (PJI) also rises [1]. The demand for TKA revision in the United States will increase until 2030 up to 673% and for THA up to 174% comparing to the cases registered in 2005 [2]. In Austria, the number of TKA and THA revisions increased from 2009 to 2015 with 5.3% for TKA (919 cases) and 7.1% for THA (1290 cases) [3]. Around 1–3% of patients undergoing TKA and THA develop PJI [1]. The pathogenesis of PJI is related to the presence of bacteria on the surface of an implant or host tissue forming a biofilm. Biofilms are well-organized communities of bacterial cells protected by a self-produced extracellular matrix, which protects the microorganisms against stress and other external factors, as well as increasing the tolerance to antibiotic substances. In addition, bacteria encased in biofilms are hard to identify by standard diagnostic procedures [4,5].

Hospitals worldwide test the antibiotic resistance of patient isolated strains following standard protocols. Examples are the European Committee on Antimicrobial Susceptibility Testing (EUCAST) and the Clinical Laboratory Standards Institute (CLSI). The objective is to investigate the minimal inhibitory concentration (MIC) and the antibiotic breakpoint (ABp) of substances taking into consideration different pathogens using different methods like broth dilution, disk diffusion or antimicrobial diffusion gradients [6].

Research laboratories that are studying biofilms use different models and culture media to elucidate phenotypic, genotypic and physiologic mechanisms of biofilm formation and antibiotic susceptibility [7,8]. Recent studies show that it is of interest to evaluate if culture media have an effect on the biofilm development. Microorganisms can present different growth pathways, antibiotic susceptibility and express different genes according to the media used [9,10]. Some even fail to develop complex biofilms depending on the in vitro set up [11].

For *Staphylococcus epidermidis*, one of the most common coagulase-negative *staphylococci* (CoNS), several proteins are essential for biofilm formation [12]. One important mechanism of biofilm formation is the production of a polysaccharide intercellular adhesion (PIA) [13]. PIA is synthetized by several enzymes and encoded by the *ica* operon, including the *ica*A, *ica*D, *ica*B and *ica*C genes [14]. Several studies showed that the *ica*ADBC genes are present in a great variety of clinically significant *S. epidermidis* strains [15,16,17].

In vitro tests with biofilms should be able to simulate the implant/host tissue interfaces. Laboratory methods, which are carried out towards this objective, are more successful in further translating the results to the clinical reality. One possibility is the use of synovial fluid (SF) as medium for cultivating biofilms in vitro. Disadvantages are related to the limitation in the obtainment of adequate amounts from patients [18]. One alternative can be the use of bovine, porcine or equine SF [19] or the use of simulated SF [20,21].

In this study, we investigated the effect of different nutrient media compared to pooled and diluted human SF on the antibiotic susceptibility of bacteria in biofilm form. In addition, we evaluated the influence of the nutrient media on expression of the *ica*ADBC genes during biofilm formation. The aim of this study was to evaluate the impact of different nutrient media on the growth, antibiotic susceptibility, cell viability and gene expression of coagulase-negative *staphylococci* (CoNS) biofilms. 

## 2. Materials and Methods

### 2.1. Strain Selection

In this study, we used five different CoNS strains. One *Staphylococcus epidermidis* resistant against gentamicin, clindamycin and rifampicin (*S. epidermidis* R), one *Staphylococcus epidermidis* susceptible to the antibiotics tested, as well as *Staphylococcus simulans*, *Staphylococcus lugdunensis* and *Staphylococcus haemolyticus* (resistant against clindamycin). The clinical isolates were obtained from patients undergoing PJI treatment at the University Hospital for Orthopaedics and Traumatology of the Medical University Innsbruck, Austria. The protocol used in this study was evaluated and approved by the Human Ethic Committee of the Medical University Innsbruck (AN2017-0072 371/4.24 396/5.11-4361A). Prior to the beginning of the tests, we inoculated the six strains on Müller-Hinton agar plates (Mueller-Hinton-Agar VM556437 329, pH 7.4 ± 0.2 at 25 °C, MERCK KGaA, Darmstadt, Germany) with a sterile loop and incubated for 72 h at 37 °C for the obtainment of fresh colonies.

### 2.2. Antibiotics Used

Gentamicin (gentamicin sulphate, Refobacin^®^ 80 mg ampoules, Merck KGaA. Darmstadt, Germany), clindamycin (clindamycin phosphate, Dalacin-C^®^ Phosphate 300 mg/2 mL, Pfizer Corporation Austria GmbH, Vienna, Austria), vancomycin (vancomycin hydrochloride, Vancocin^®^ 500 mg, Astro Pharma GmbH, Vienna, Austria) and rifampicin (Rifoldin 600 mg, SANOFI-AVENTIS GmbH, Vienna, Austria) were used. We based the choice of concentration used in this study on the results obtained from minimal inhibitory concentration (MIC) and biofilm inhibitory concentration (BIC) tests carried out in advance. For the tests, we chose for each antibiotic substance a concentration between MIC and BIC.

### 2.3. Nutrient Media

We used Tryptic Soy Broth (TSB, Merck KGaA, Darmstadt, Germany) and Müller-Hinton Bouillon (MHB, Merck KGaA, Darmstadt, Germany) pure and supplemented with 1% glucose (TSB+G/MHB+G). For the biofilm inhibitory concentration (BIC) and the recovery test, MH agar plates were used. Human synovial fluid (SF) was obtained from several non-infected patients, pooled and diluted 1:4 in phosphate buffered saline (PBS, 9.55 g/L Dulbecco, Biochrom GmbH, Berlin, Germany). The diluted SF was used for all experiments. For comparison reasons, we also used SF supplemented with 1% glucose (SF+G). The use of the patient’s joint aspirate was only carried out after the obtainment of the patient’s consent. The protocol used for the collection of joint aspirates was evaluated and approved by the Human Ethic Committee of the Medical University Innsbruck (AN2017-0072 371/4.24 396/5.11-4361A).

### 2.4. Gene Expression Analysis

For the RNA isolation we used the Qiagen RNeasy Mini Kit (RNeasy^®^ Mini Kit, Qiagen GmbH, Hilden, Germany). DNase treatment was performed with DNase I (DNase I RNase-free, 2000 U/mL, New England Biolabs, Frankfurt am Main, Germany). For the cDNA synthesis we used the iScript^TM^ reverse transcription Supermix for RT-qPCR (iScript^TM^ RT Supermix, Bio-Rad Laboratories, Feldkirchen, Germany) and the PicoReal 96 System (PikoReal 96 Realtime PCR System, Thermo Fisher Scientific, Waltham, MA, USA) for the thermo cycling. The real-time qPCR was performed with the iQ^TM^ SYBR Green Supermix (iQ^TM^ SYBR Green Supermix, Bio-Rad Laboratories, Feldkirchen, Germany) and the PicoReal 96 System (PikoReal 96 Realtime PCR System, Thermo Fisher Scientific, Waltham, MA, USA).

### 2.5. Primers for Biofilm Genes Used in RT-qPCR

Table 1 shows the primers used for the RT-qPCR. Primers were synthetized by Metabion (Metabion GmbH, Planegg/Steinkirchen, Germany). The lyophilized primers were diluted to 100 µM following the manufacturer’s recommendations. Primers were then diluted to 10 µM stock solutions for further use. For the two *S. epidermidis* strains the primers were synthetized based on the references mentioned in Table 1. For the other strains the corresponding gene sequences (Table 2) were acquired from GeneBank and primers were designed by Primer-Blast (National Center for Biotechnology Information—NCBI, Bethesda MD, MD, USA) [22]. 

### 2.6. Initial Biofilm Formation

With the aim to have the same starting conditions for each strain for further treatment with different media and antibiotics, we carried out the initial biofilm formation using only TSB+G. For the preparation of the inoculum for the biofilm formation, we suspended three colonies of each strain in 2 mL TSB+G in a 15 mL centrifuge tube (VWR International, Radnor, PA, USA) and incubated at 37 °C in a moist chamber under constantly circular shaking (Edmund Bühler GmbH, Hechingen, Germany) at 200 rpm for 24 h. After incubation, we diluted the bacterial solutions in fresh TSB+G to a concentration of 10^5^ bacteria/mL. We used conventional 96-well plates (Corning^®^, Amsterdam, The Netherlands) for the biofilm formation. For that we added 100 µL of the inoculum in each well containing a sterile stainless-steel disc (DIN9021, stainless steel A2, size M2, diameter 5.9 mm) which was used as a substrate. The 96-well plates were incubated in a moist chamber under constant circular shaking at 37 °C for 48 h.

### 2.7. Determination of the Minimal Inhibitory Concentration (MIC) 

The MIC was determined using *E*-test stripes (bioMérieux Austria GmbH, Vienna, Austria). The bacterial strains were inoculated on MH agar plates with a sterile loop. Afterwards we placed gentamicin, clindamycin, vancomycin and rifampicin *E*-test strips on the plates inoculated with each strain. The plates were incubated at 37 °C for 24 h. After incubation, we measured the zones of inhibition for the determination of the MIC.

### 2.8. Determination of the Biofilm Inhibitory Concentration (BIC)

For the determination of the BIC, biofilm was formed on sterile metal discs for 48 h (see Section 2.6). For the susceptibility tests, antibiotic solutions were prepared using TSB+G in 12 different concentrations (0.5, 1, 2, 4, 8, 16, 32, 64, 128, 256, 512 and 1024 µg/mL) of each substance. Untreated discs with biofilm were used as control. After the biofilm formation, we washed the discs with sterile PBS for the removal of the planktonic bacteria and treated with 100 µL of TSB+G containing the antibiotics. We incubated the plates containing the treated biofilms at 37 °C in a moisturized chamber under constantly circular shaking for 24 h. After 24 h, the discs were washed again in sterile PBS and added into 2 mL micro centrifuge tubes (VWR International, Radnor, PA, USA) containing 1 mL of fresh PBS. The tubes were sonicated for 5 min on 100% intensity (ultrasonic peak power: 800 W; Bactosonic, Bandelin electronic GmbH and Co. KG, Berlin, Germany). The fluid obtained after sonication, containing the detached biofilms, was diluted 1:100 in PBS and 10 µL inoculated on MH agar plates with a sterile loop. After inoculation we incubated the plates at 37 °C for 24 h. 

### 2.9. Antibiotic Susceptibility Tests with Different Nutrient Media

After the obtainment of biofilm (see Section 2.6), we washed the discs with sterile PBS for the removal of the planktonic bacteria and placed them in wells containing 100 µL of TSB, TSB+G, MHB, MHB+G, SF or SF+G containing the concentrations of the antibiotics described in Table 4. The 96-well plates containing the biofilms, media and antibiotics were placed at 37 °C in a moist chamber under constant circular shaking for 24 h. An untreated sample was made for control of the antibiotic activity.

### 2.10. Evaluation of Antibiotic Activity in Different Nutrient Media by Measuring Biofilm Recovery Capacity 

We counted the colony forming units (CFUs) of the recovered bacteria to determine the antibiotic activity of each substance tested in different media. Discs with biofilm and no treatment were used as controls. After the treatments we washed the discs in sterile PBS for the removal of planktonic bacteria and added them into micro centrifuge tubes containing 1 mL fresh PBS. We sonicated the tubes for 3 min in an ultrasound bath with 100% intensity (ultrasonic peak power: 800 W; Bactosonic, Bandelin electronic GmbH and Co. KG, Berlin, Germany). The fluid obtained after sonication, containing the detached biofilms, was diluted 1:100 in PBS. We inoculated 50 µL of the sonication fluid on MH ager plates with an automatic spiral plater (model WASP2, DonWhitley Scientific, Shipley, UK). After inoculation, we incubated the plates at 37 °C for 12–24 h. After incubation, we counted the CFUs.

### 2.11. Evaluation of Antibiotic Activity in Different Nutrient Media by Measuring Bacterial Viability

After antibiotic treatment we evaluated the viability of the remaining cells using tetrazolium salt reduction and optical density (OD). For that we washed the discs in sterile PBS and placed them into a new 96-well plate, each well containing 100 µL PBS. Prior to the experiments XTT solution was prepared by mixing 1 mL sodium 3′-[1-(phenylaminocarbonyl)-3,4-tetrazolium]-bis (4-methoxy-6-nitro) benzene sulfonic acid hydrate (XTT labeling reagent, Cell Proliferation Kit II (XTT), Sigma-Aldrich, St. Louis, MO, USA) with 20 µL N-methyl dibenzopyrazine methyl sulfate (PMS, Electron coupling reagent, Cell Proliferation Kit II (XTT), Sigma-Aldrich, St. Louis, MO, USA). Following, we added 50 µL of the XTT solution into each well. Two wells were used as a control (ODc) without disc and biofilm. We incubated the plates at 37 °C in the dark using a moist chamber under constant circular shaking (Edmund Bühler GmbH, Hechingen, Germany). After 3 h incubation, 100 µL of each well were transferred to a new plate and measured at 450 nm in a photometric reader (Multiskan FC, Thermo Fisher Scientific, Waltham, MA, USA). To remove eventual backgrounds, the mean of the ODc was then subtracted from each value.

### 2.12. Isolation of Bacterial RNA from Biofilm Grown in Different Nutrient Media on Stainless Steel Discs

For the isolation of bacterial RNA, biofilm was grown for 48 h (see Section 2.6). Differently from the protocol used for biofilm recovery and cell viability, here the biofilm was grown in each different medium from the beginning to investigate its influence on the gene expression. In order to increase the area of biofilm formation and to obtain the sufficient amount of RNA for the tests, the biofilms were grown using a pool of 10 discs for each strain in each nutrient medium.

Following the biofilm formation, the discs containing the biofilm were washed in sterile PBS for the removal of the planktonic bacteria. The discs were put into a 1.5 mL micro centrifuge tube and 1 mL of TRI-Reagent (TRI Reagent^®^, Sigma-Aldrich, St. Louis, MO, USA) was added. The tubes were vortexed for 15 s and sonicated for 3 min in an ultrasound bath with 100% intensity (ultrasonic peak power: 800 W; Bactosonic, Bandelin electronic GmbH & Co. KG, Berlin, Germany). The liquid inside was transferred into 2 mL screw cap micro tubes (Screw cap micro tubes, Sarstedt AG and Co., Nümbrecht, Germany) containing 25–50 mg of glass beads (acid-washed glass globules, Ø 0.1 mm, Carl-Roth GmbH + Co. KG, Karlsruhe, Germany). The tubes were added into a FastPrep-24^TM^ 5G (MP Biomedicals, Thermo Fisher Scientific, Waltham, MA, USA) and processed three times for 35 s at 10 m/s. In between the repetitions, the tubes were kept on ice for 2 min. After the third repeat, 200 µL chloroform was added. The tubes were vortexed for 15 s and then incubated at room temperature for 7 min. 

After incubation, the tubes were centrifuged at 4 °C and 12,000 G for 15 min. Afterwards, the upper phase containing the RNA was collected and transferred to a 1.5 mL micro centrifuge tube. An equal volume of 70% ice cold ethanol was added and gently mixed using a pipette. The manufacturer’s procedures described in the QIAGEN Supplementary Protocol. Purification of total RNA from bacteria using RNeasy^®^ Mini Kit were used with some adaptations. The liquid for each strain was pooled on one membrane provided by the manufacturer. For the elution step, 50 µL of RNase-free water were used. Afterwards, 1µL of the solution was analyzed with a spectrophotometer (DeNovix DS-11 FX +µVolume Spectrophoto-/Fluorometer, Biozym Scientific GmbH, Hessisch Oldendorf, Germany) for RNA quantification. After measurement, the RNA was treated with DNase I. RNase-free water and 10 µg of RNA were mixed together to achieve a total volume of 25 µL. Afterwards, we added and mixed 1 µL of DNase I (2 units) with 2.6 µL of 10×Buffer from the kit. The mixture was first incubated at 37 °C for 30 min. After the incubation, EDTA was added to inactivate the reaction with a final concentration of 5 mM and incubated at 75 °C for 10 min. After DNase treatment, 1 µL RNA was measured with a spectrophotometer. 

### 2.13. Synthesis of cDNA from Bacterial RNA

Following the isolation of bacterial RNA and DNase I treatment, 1 µg of RNA was used for cDNA synthesis following the procedures suggested by the manufacturer (iScript^TM^ RT Supermix, Bio-Rad Laboratories, Feldkirchen, Germany). The reaction protocol was carried out using the PikoReal 96 System (PikoReal 96 Realtime PCR System, Thermo Fisher Scientific, Waltham, MA, USA).

### 2.14. Investigation of the Influence of Different Nutrient Media on the Bacterial Biofilm Gene Expression Using Real-Time Quantitative Polymerase Chain Reaction (Real-Time qPCR)

After obtainment of the cDNA, the protocol of the iQ^TM^ SYBR Green Supermix kit (iQ^TM^ SYBR Green Supermix, Bio-Rad Laboratories, Feldkirchen, Germany) was followed and the tests proceeded with a total volume of 20 µL per reaction. For the real-time qPCRs, we used 250 ng of each forward and reverse primer added to 50 ng of cDNA. For the thermal cycling protocol, we used the same temperatures, but maximum times mentioned in the manufacturer’s procedures (iQ^TM^ SYBR Green Supermix, Bio-Rad Laboratories, Feldkirchen, Germany). The melting curve analysis was performed with the instrument default settings (PikoReal 96 Realtime PCR System, Thermo Fisher Scientific, Waltham, MA, USA).

### 2.15. Data Analysis

All results were analyzed using GraphPad Prism 7 (GraphPad Software, Inc., La Jolla, CA, USA). For the statistical analysis of CFU and viability data, we used two-way analysis of variance (ANOVA) with Dunnett’s and Turkey’s multiple comparison tests taking into consideration the different antibiotics and media used. For the analysis of gene expression, we used two-way/one-way analysis of variance (ANOVA) with Dunnett’s/Sidak’s multiple comparison test taking into consideration the different nutrient media used. *p* < 0.05 was considered to be statistically significant. Further statistical data are displayed in Supplementary Tables S1–S5.

The threshold for the determination of the Ct values was automatically set by the equipment. The results for the bacterial gene expression were calculated by the 2^−ΔCt^ method. All real-time qPCR results were normalized to the gene expression of 16S rRNA. 

## 3. Results

### 3.1. Identification of the MIC and BIC

The results of the MIC and BIC showed, for all strains and antibiotics, that the BIC exceeded the MIC values (Table 3). As expected, the MIC from the *S. epidermidis* strain resistant to gentamycin, clindamycin and rifampicin showed higher MIC values. For all strains, except the *S. epidermidis* R and the *S. haemolyticus*, vancomycin showed the least effect, whereas rifampicin had the best effect, except for *S. epidermidis* R. For almost all strains and antibiotics, the BIC value was over 1024 µg/mL. As the aim of this study was to evaluate the impact of the nutrient media on the antibiotic susceptibility, it was important to use concentrations which were not able to eliminate the biofilms completely. The concentrations we used are highlighted in Table 4.

### 3.2. Recovery of Biofilm Cells after Treatment with Different Antibiotics Using Different Nutrient Media

**Figure 1:**
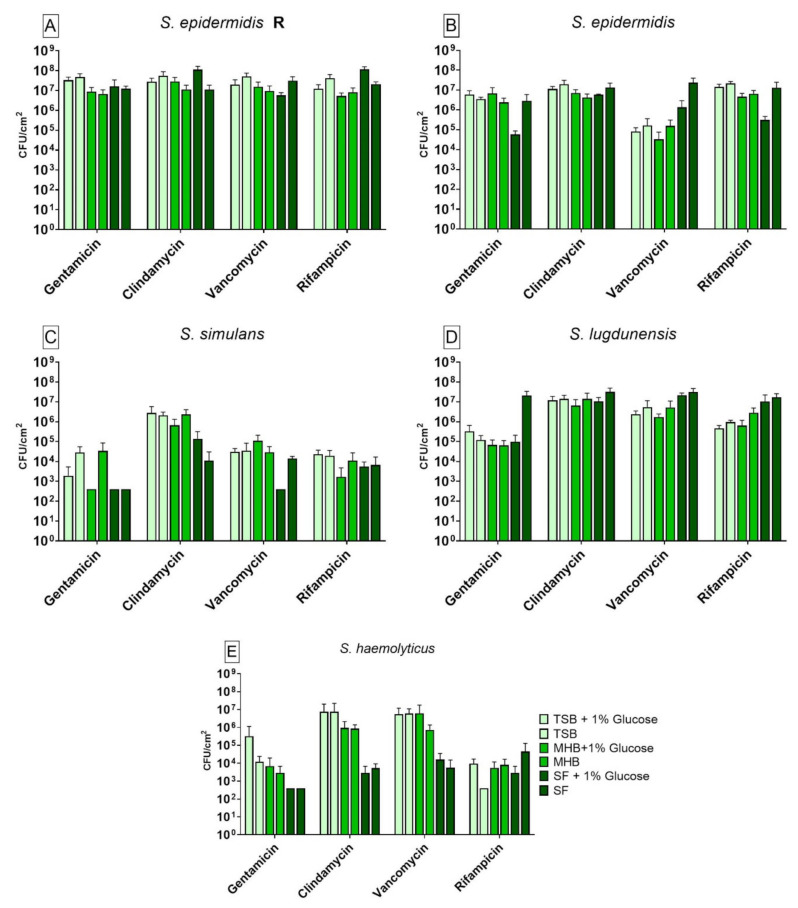
Results of the colony forming units (CFU) counting after antibiotic treatment with gentamicin, clindamycin, vancomycin and rifampicin for biofilm produced by *S. epidermidis* R (**A**), *S. epidermidis* (**B**), *S. simulans* (**C**), *S. lugdunensis* (**D**) and *S. haemolyticus* (**E**) in different nutrient media. The differences to biofilm grown in SF were analyzed with two-way ANOVA with Tukey’s multiple comparison tests. The difference between each nutrient medium with its glucose alternative was analyzed with two-way ANOVA with Dunnett’s multiple comparison tests. The mean values of three different experiments performed in triplicates +SD are shown. Detection limit was 398 CFU/cm^2^.

We observed significant differences on the bacterial recovery of biofilms between antibiotics and nutrient media used. When compared to SF, the *S. epidermidis* R strain showed significant differences in CFU counting for gentamicin, clindamycin, vancomycin and rifampicin (Figure 1A). A difference in biofilm cell recovery between each medium and its variant with 1% glucose could be observed with TSB when treated with rifampicin and with SF when treated with clindamycin, vancomycin and rifampicin. Biofilms incubated in TSB showed significant higher cell recovery when treated with rifampicin compared to SF. The biofilm grown in TSB and TSB+G showed a higher tolerance to gentamicin and clindamycin when compared to biofilm grown in SF. Biofilm formed in MHB+G showed a significant difference in the susceptibility to clindamycin and rifampicin when compared to SF. Its alternative without glucose, MHB, showed a significant lower tolerance against vancomycin and rifampicin when compared to biofilm grown in SF. The samples treated with clindamycin and rifampicin showed a higher biofilm cell recovery in SF+G compared to SF. Furthermore, biofilm grown in TSB+G and treated with rifampicin showed a significantly lower tolerance to the antibiotic than its counterpart without glucose. Only biofilm treated with vancomycin showed lower biofilm cell recovery in SF+G in comparison to SF. The susceptible form of *S. epidermidis* (Figure 1B) also showed differences in recovery after antibiotic treatment using different media. Differences could be observed for treatment with gentamicin, vancomycin and rifampicin when compared to the results for biofilm cell recovery in SF. Only a treatment with clindamycin was not able to reduce the cell counting significantly. Additionally, glucose did have a significant effect on biofilm grown in MHB+G for vancomycin and in SF+G for gentamicin, vancomycin and rifampicin. When treated with gentamicin, biofilm grown in TSB+G and MHB+G showed a significantly higher tolerance to the antibiotic than biofilm cultivated in SF. For biofilm treated with vancomycin, all media showed a significantly lower biofilm recovery than biofilm grown in SF. When compared with biofilm grown in SF, biofilm produced in SF+G showed a lower recovery when treated with gentamicin, vancomycin and rifampicin. Additionally, biofilm grown in MHB showed a higher recovery when treated with vancomycin compared to biofilms grown in MHB+G. The results for *S. simulans* are shown in Figure 1C. Detection limit (398 CFU/cm^2^) was reached for gentamicin with MHB+G, SF+G and SF and for vancomycin with SF+G. A different susceptibility to antibiotics was observed for several nutrient media with treatment with gentamicin, clindamycin and vancomycin when compared to the results for biofilm recovery in SF. The influence of glucose on biofilm recovery could be observed with TSB/TSB+G for gentamicin and SF/SF+G for clindamycin and vancomycin. The activity of clindamycin on biofilm varied between the media used when compared to SF. We observed a significant higher tolerance to gentamicin for biofilm cultivated in TSB and MHB when compared to biofilm from SF. For the treatment with clindamycin all other media showed a higher tolerance when compared to biofilm grown in SF. When compared to the variant with 1% glucose, TSB showed a higher tolerance to gentamicin. Furthermore, biofilm grown in SF had a different tolerance to clindamycin and vancomycin than its counterpart with glucose. When compared to biofilm grown in SF, the *S. lugdunensis* biofilms (Figure 1D) showed significant differences in susceptibility for treatment with gentamicin, clindamycin, vancomycin and rifampicin for several nutrient media. Glucose only showed an effect when added to SF and treated with gentamicin. The strain showed higher tolerance to gentamicin and clindamycin when cultivated in SF in contrast to biofilm cultivated in the other media. When treated with vancomycin and rifampicin, all nutrient media except SF+G showed a significantly lower biofilm cell recovery when compared to SF. When compared to the glucose alternative, SF showed a higher tolerance to gentamicin. Biofilm recovery rate by *S. haemolyticus* (Figure 1E) was significantly different among the media used. Detection limit (398 CFU/cm^2^) was reached for gentamicin with SF+G and SF and for rifampicin with TSB. Different susceptibility to antibiotics was observed for treatment with all antibiotics when compared to biofilm grown in SF. No significant differences could be observed between each medium and its variant with 1% glucose. For the treatment with gentamicin and rifampicin, only biofilm grown in TSB showed a significantly higher tolerance than biofilm grown in SF. When treated with clindamycin and vancomycin, biofilm in all other media except SF+G had a higher tolerance to the antibiotics than biofilm formed in SF. 

### 3.3. Bacterial Viability after Treatment with Different Antibiotics Using Different Nutrient Media

**Figure 2:**
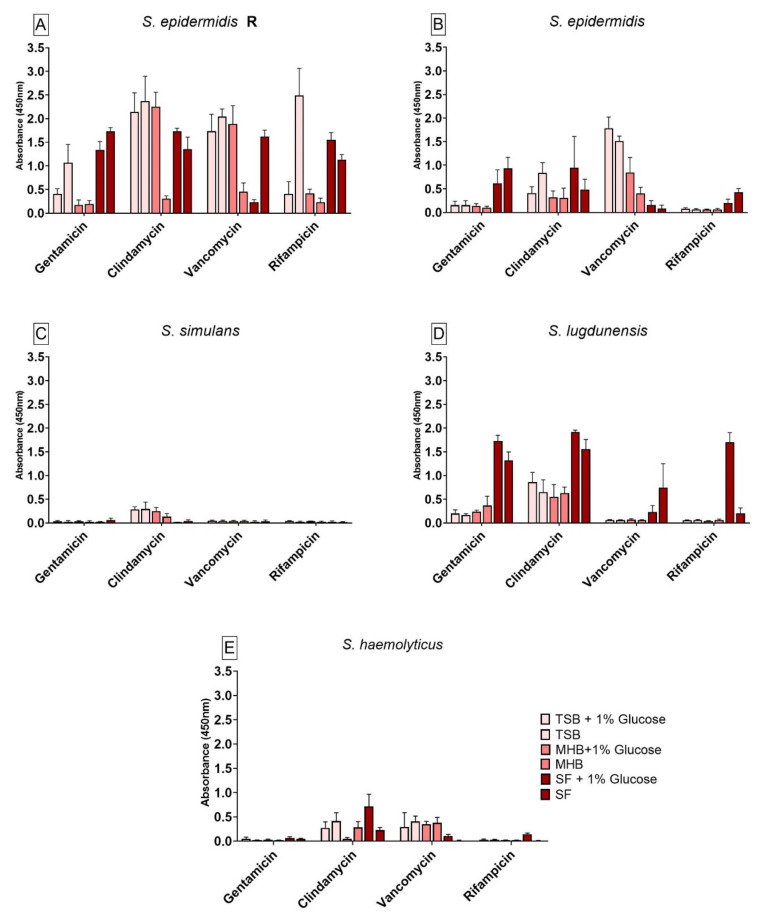
Results of the cell viability test (XTT) for biofilm produced by *S. epidermidis* R (**A**), *S. epidermidis* (**B**), *S. simulans* (**C**), *S. lugdunensis* (**D**) and *S. haemolyticus* (**E**) after antibiotic treatment in different nutrient media. The differences to biofilm grown in SF were analyzed with two-way ANOVA with Tukey’s multiple comparison tests. The difference between each nutrient medium with its glucose alternative was analyzed with two-way ANOVA with Dunnett’s multiple comparison tests. The mean values of three different experiments performed in triplicates +SD are shown.

The results for the viability test for all six CoNS are shown in Figure 2. All six strains showed significant differences in cell viability after antibiotic treatment between the different media when compared to SF. Glucose seemed to have an effect on the antibiotic susceptibility. *S. epidermidis* R biofilm (Figure 2A) showed high cell viability over all other strains after the antibiotics tested. Only a cultivation in MHB+G and MHB seemed to have a negative impact on the antibiotic susceptibility when compared to SF. As expected, the susceptible form of *S. epidermidis* strain (Figure 2B) showed less cell viability after antibiotic treatment than the *S. epidermids* R. When cultivated in TSB+G, TSB and MHB+G, the biofilm bacteria had a higher cell viability after antibiotic treatment with vancomycin when compared to SF. With a treatment with gentamicin and rifampicin, the cultivation in SF and SF+G seemed to increase the cell viability after antibiotic treatment. When treated with clindamycin and rifampicin, glucose had a significant effect on the antibiotic susceptibility for biofilm cultivated in all nutrient media compared to their counterparts without glucose. The cell viability of *S. simulans* (Figure 2C) biofilm showed very low absorbance readings. No significant differences in cell viability when treated with gentamicin, vancomycin and rifampicin could be observed when compared to biofilm grown in SF. When treated with clindamycin, a higher cell viability of biofilm could be achieved by cultivation in TSB+G, TSB and MHB+G when compared to biofilm grown in SF. The addition of glucose did not have a significant influence on the antibiotic susceptibility for this strain. The cell viability of biofilm produced by *S. lugdunensis* (Figure 2D) showed significant differences after antibiotic treatment with gentamicin, clindamycin, vancomycin and rifampicin when compared to biofilm formed in SF. The cell viability in SF and SF+G was the highest over all other nutrient media used. Glucose showed a significant influence on the viability of the bacteria during treatment with all antibiotics in SF when compared to SF+G. The cell viability of biofilm produced by *S. haemolyticus* (Figure 2E) showed significant differences when treated with clindamycin, vancomycin and rifampicin compared to biofilm grown in SF. Biofilm cultivated in SF and treated with vancomycin had a significantly lower cell viability than biofilm grown in all other nutrient media. Furthermore, biofilm cultivated in SF had a lower cell viability than biofilm formed in SF+G when treated with clindamycin, vancomycin and rifampicin.

### 3.4. Impact of Different Nutrient Media and Glucose on the Biofilm Growth

**Figure 3:**
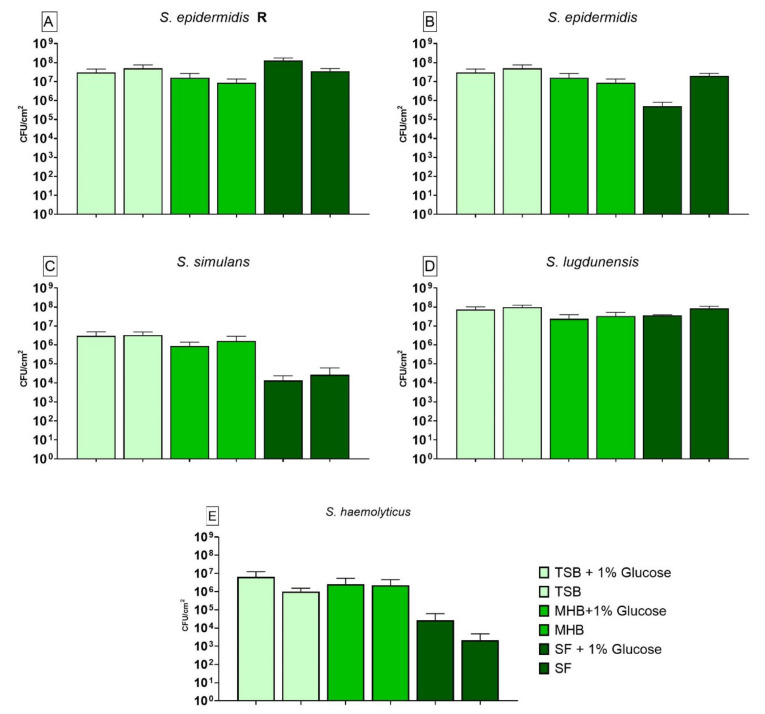
Results of the colony forming units (CFU) counting for biofilm produced by *S. epidermidis* R (**A**), *S. epidermidis* (**B**), *S. simulans* (**C**), *S. lugdunensis* (**D**) and *S. haemolyticus* (**E**) in different nutrient media. The differences to biofilm grown in SF were analyzed with two-way ANOVA with Tukey’s multiple comparison tests. The difference between each nutrient medium with its glucose alternative was analyzed with two-way ANOVA with Dunnett’s multiple comparison tests. The mean values of three different experiments performed in triplicates +SD are shown. Detection limit was 398 CFU/cm^2^.

The results of the CFUs counting for biofilm grown in different nutrient media without any treatment are shown in Figure 3. The *S. epidermidis* R (Figure 3A) strain showed significantly lower growth when cultivated in MHB compared to biofilm grown in SF. Glucose only had a positive effect on the biofilm formed in SF+G when compared to its alternative without glucose. Biofilm produced by *S. epidermidis* (Figure 3B) only showed significantly lower growth between SF and SF+G whereas biofilm formed by *S. simulans* (Figure 3C) showed significantly higher growth when cultivated in TSB+G, TSB, MHB+G and MHB and compared to biofilm produced in SF. Glucose led to no significant difference for this strain when compared to each medium without glucose. *S. lugdunensis* biofilm (Figure 3D) did not show any significant differences in growth when compared to biofilm grown in SF. Glucose also had no effect on the biofilm formation. For *S. haemolyticus* (Figure 3E) all nutrient media tested had a significantly higher biofilm growth when compared to biofilm grown in SF. Glucose led to a significantly higher cell count when biofilm was grown in SF+G.

### 3.5. Impact of Different Nutrient Media and Glucose on the Biofilm Cell Viability

**Figure 4:**
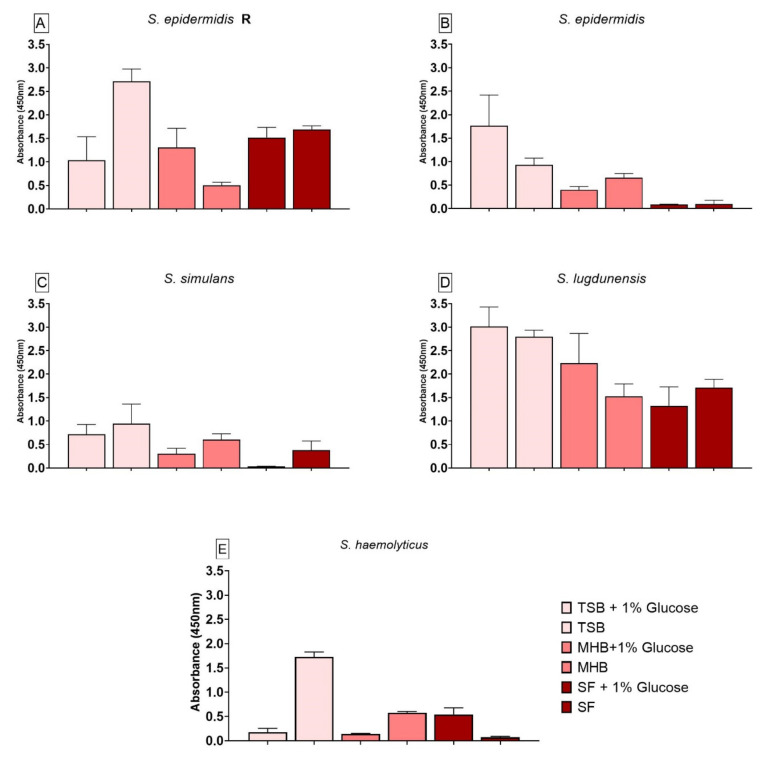
Results of the cell viability test (XTT) for biofilm produced by *S. epidermidis* R (**A**), *S. epidermidis* (**B**), *S. simulans* (**C**), *S. lugdunensis* (**D**) and *S. haemolyticus* (**E**) in different nutrient media. The differences to biofilm grown in SF were analyzed with two-way ANOVA with Tukey’s multiple comparison tests. The difference between each nutrient medium with its glucose alternative was analyzed with two-way ANOVA with Dunnett’s multiple comparison tests. The mean values of three different experiments performed in triplicates +SD are shown.

Biofilm produced by *S. epidermidis* R (Figure 4A) showed significantly lower cell viability when grown in TSB+G and MHB, whereas biofilm formed in TSB had a significantly higher cell viability when compared to SF. The presence of glucose in the media had a significant effect on biofilm grown in TSB+G and MHB+G. Biofilm formed by *S. epidermidis* (Figure 4B) also showed significant differences in viability when compared to biofilm grown in SF. Biofilm produced in TSB+G, TSB and MHB had a significantly higher cell viability than biofilm produced in SF. The cell viability in TSB+G was significantly higher when compared to biofilm grown in TSB. *S. simulans* biofilm (Figure 4C) had a higher cell viability when formed in TSB+G, TSB and MHB when compared to biofilm produced in SF. For this strain, media enriched with glucose showed decreased cell viability in comparison to the variants without glucose (TSB, MHB and SF). Biofilm produced by *S. lugdunensis* (Figure 4D) and cultivated in SF had a significantly lower cell viability than biofilm grown in TSB+G, TSB, MHB+G. A significant positive influence of glucose could only be observed for MHB+G. The cell viability of biofilm produced by *S. haemolyticus* (Figure 4E) grown in SF was significantly lower than cell viability of biofilm formed in TSB and MHB. The addition of glucose showed an effect on all different media. For TSB+G and MHB+G it decreased the viability significantly whereas for SF+G it led to a higher cell viability.

### 3.6. Influence of Different Nutrient Media and Glucose-Enriched Nutrient Media on the Bacterial icaADBC Gene Expression Compared to Biofilm Grown in SF

**Figure 5:**
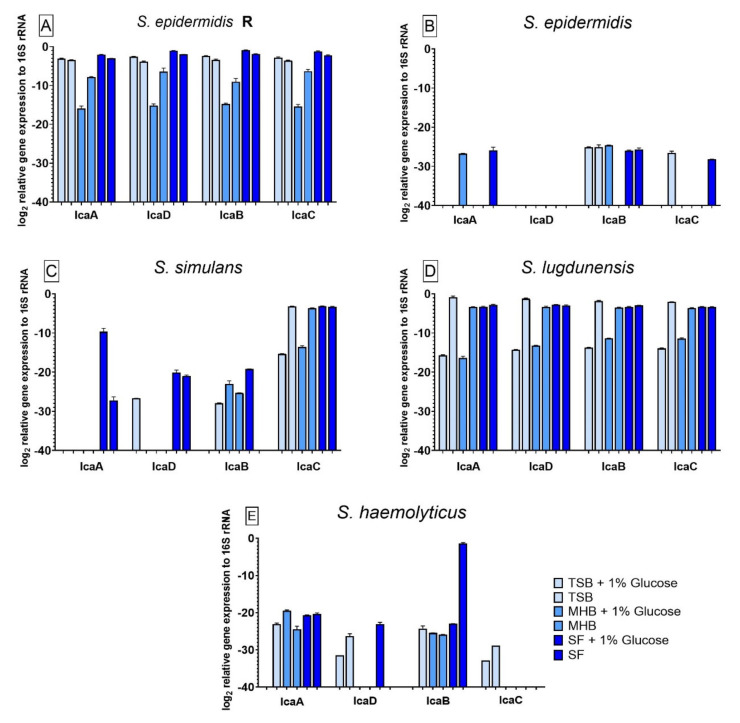
Results of the *ica*ADBC gene expression for biofilm produced by *S. epidermidis* R (**A**), *S. epidermidis* (**B**), *S. simulans* (**C**), *S. lugdunensis* (**D**) and *S. haemolyticus* (**E**) in different nutrient media. The results for the comparison between the nutrient media and SF were analyzed with one-way ANOVA with Dunnett’s multiple comparison tests. The difference between each nutrient medium with its glucose alternative was analyzed with one-way ANOVA with Sidak’s multiple comparison tests. The log_2_ mean values of three different experiments performed in triplicates +SD are shown. No bar is equivalent to no gene expression.

All strains tested showed significant differences in *ica*ADBC gene expression when compared to biofilm grown in SF. Additionally, the presence of glucose in the media had significant effects on the gene expression for all strains. The relative gene expression in *S. epidermidis* R (Figure 5A) was significantly lower for all tested genes which were expressed by biofilm growing in TSB, MHB+G and MHB in comparison to SF. For biofilm grown in MHB+G the expression decreased significantly when glucose was added. For *S. epidermidis* (Figure 5B) *ica*A was not expressed in TSB+G, TSB, MHB and SF+G. No gene expression could be observed for *ica*D in all nutrient media. *Ica*B was not expressed when biofilm was grown in TSB pure and the expression of *ica*C was not observed in biofilm cultivated in TSB+G, MHB+G, MHB and SF+G. When compared to the gene expression in SF, the different nutrient media led to a significantly lower expression of *ica*A in each medium except MHB+G. For *ica*B, cultivation in MHB+G led to a lower, and cultivation in MHB to a higher expression. The expression of *ica*C was significantly lower in all nutrient media except TSB, where it was higher. Effects of glucose could be observed for *ica*A in MHB+G and SF+G, for *ica*B in MHB+G and for *ica*C in TSB+G and SF+G. Gene expression from *S. simulans* biofilm (Figure 5C) showed significant influence of glucose-enrichment and the different nutrient media compared to SF on all genes. No expression could be observed for *ica*A in TSB+G, TSB, MHB+G and MHB, for *ica*D in TSB, MHB+G and MHB and for *ica*B in TSB+G and SF. For *ica*A and *ica*D, biofilm grown in SF had significant different gene expression than biofilm grown in all other media. In contrast to the non-detection of *ica*B expression in biofilm formed in SF, biofilm from all other media, except TSB+G, showed significantly high expression. Gene expression of *ica*C was significantly lower than the expression in SF when biofilm was cultivated in TSB+G and MHB+G. Glucose-enrichment promoted a significantly higher expression of *ica*A in SF+G than in SF. Expression of *ica*B was detectable in TSB+G compared to the missing expression in TSB. The addition of glucose improved the expression of *ica*B in SF+G compared to its non-detection in SF. Compared to the variant with 1% glucose, gene expression of *ica*C in TSB and MHB was significantly higher. The gene expression for *S. lugdunensis* biofilms (Figure 5D) also showed significant differences when compared to gene expression in SF. For all genes, the cultivation of biofilm in TSB+G and MHB+G led to significantly lower gene expression whereas the cultivation in TSB led to a significantly higher expression of all genes when compared to biofilm grown in SF. Biofilm formed in MHB only showed a significantly lower expression of *ica*B when compared to SF. The effect of glucose on *S. lugdunensis* could be seen as a decrease of gene expression for all genes in TSB+G and MHB+G. Biofilm formed by *S. haemolyticus* (Figure 5E) showed no gene expression of *ica*A in TSB+G, of *ica*D in MHB+G, MHB and SF, of *ica*B in TSB+G and of *ica*C in all nutrient media except TSB+G and TSB. The expression of *ica*A was significantly lower in TSB+G, TSB and MHB when compared to SF. The expression of *ica*D was significantly higher in TSB+G and TSB. All nutrient media facilitated a significantly higher expression of *ica*B except TSB+G when compared to biofilm grown in SF. Only biofilm grown in TSB+G and TSB expressed *ica*C significantly higher than biofilm produced in SF. The presence of glucose might have a negative effect on the expression of all genes in TSB+G and for *ica*B in SF+G. Compared to the variants without glucose, a positive effect of glucose could be observed for *ica*A in MHB+G and *ica*D and *ica*C in SF+G.

## 4. Discussion

In this study, we investigated the effect of different nutrient media and glucose-enriched nutrient media on the biofilm formation and biofilm antibiotic susceptibility as well as on the *ica*ADBC gene expression using six different coagulase-negative *staphylococci* strains. Research and routine laboratories all over the world use a variety of nutrient media for in vitro tests and susceptibility tests of antimicrobial substances. To the best of our knowledge, the impact of the different nutrient media and media additives on the growth, gene expression and susceptibility of PJI isolated strains are not well investigated. As nutrient media, we used TSB and MHB. In addition, we used TSB and MHB supplemented with 1% glucose [27]. The enrichment of media with glucose is common practice in microbiology and cell culture, used for high throughput and optimization of laboratorial tests [28,29]. With the aim to simulate the environment of a tissue/implant interface in an articulation, we also used human SF as culture media. Due to the limitations for the obtainment of high amounts of human SF, the joint aspirates used in this study were diluted 1:4 in PBS. The SF was also investigated pure and supplemented with 1% of glucose. 

For all six strains tested, significant differences in antibiotic susceptibility, biofilm cell recovery and bacterial viability were observed after different antibiotic treatment between the nutrient media used. In general, the bacterial cell viability was lower in the nutrient media compared to SF. These different susceptibility rates for the antibiotics according to the media used might have a correlation to the activity of the antibiotics or their solubility in the different nutrient media, like described by Nayak and colleagues using vancomycin against *Enterococcus* sp. [30]. Without any antimicrobial treatment, TSB and MHB had a positive effect on the growth of bacterial biofilm for all strains when compared to SF. For *S. epidermidis* R the cultivation in TSB enhanced not only the viability, but also the tolerance to clindamycin, vancomycin and rifampicin. The same strain showed lower cell viability and higher susceptibility to all antibiotics once cultivated in MHB. The expression of the *ica*ADBC loci genes was lower in the conventional media TSB and MHB when compared to the strains cultivated in SF. 

Undeniably, the nutrition plays an important role in the bacterial growth, viability and gene expression. Standard laboratorial nutrient media are designed to enable a steady and fast growth of bacteria whereas human body fluids are lacking these additives. TSB contains peptone from casein and soymeal, D(+)-glucose monohydrate, NaCl and KH_2_PO_4_. MHB is composed of beef infusion, starch and peptone from casein. These additives like peptone are not present in the human joint fluids what lead us to question the reasonability of the use of artificial media at all. Clearly such media are used aiming at the high throughput tests, but they are too far from the reality of the tissue surroundings and the results obtained may be not optimally translated to the clinical setups. SF contains several nutrient factors, which laboratorial nutrient media do not have. One of these factors is hyaluronic acid (HA). HA is a glycosaminoglycan and a main component of the mammalian extracellular matrix [31]. Other properties, like its viscosity, a certain amount of serum proteins, human body cells or uric acid and glucose also lead to a difference in nutrient availability for bacteria [32]. As it is demonstrated by Ibberson and collaborators, the availability of different substances has an essential role on the biofilm formation. They demonstrated that HA is an important component of the biofilm matrix of *S. aureus* in PJI [31]. In nutrient media for laboratorial tests, HA is usually not used as an additive. Although no chemical analysis has been done for the SF used in this study, it is considered that HA is present in the human SF with 1–4 mg/mL. This could lead to a significant impact on the biofilm formation in a human joint environment [33]. Our results also demonstrate that the cultivation of biofilm in SF or SF+G led to a significant difference in growth, viability, antibiotic susceptibility and gene expression. 

Glucose, used as an additive, enhances bacterial growth by providing more energy sources [34]. It is also a marker for hyperglycemia, which is associated with type 2 diabetes mellitus [35]. SF of healthy patients contains around 95–100% of the blood glucose level [32]. Due to this correlation, hyperglycemia could lead to a higher level of glucose in the SF and so diabetes mellitus becomes an independent risk factor for nosocomial implant infections or PJI [36,37]. For *Escherichia coli,* for instance, it could be demonstrated that a high glucose concentration leads to a higher tolerance to antibiotics [38]. We used the glucose-enriched media in our study because it is routinely used in biofilm research and routine laboratories. The aim here was to investigate its effect as a growth booster on the bacterial antimicrobial susceptibility, biofilm formation and expression of biofilm-related genes. In addition, the information obtained here can further be used for the investigation of biofilm-related infections in diabetic patients. In this study, the antibiotic susceptibility was higher when glucose was added to the nutrient medium, but it had an equal effect on the viability of cells after antibiotic treatment. Without any antimicrobial treatment, glucose seemed to have a negative effect on the cell viability as well as on the *ica*ADBC gene expression. A possible explanation for this could be that when glucose is added to a nutrient medium, the bacteria can gain more energy through the use of glucose and do not need to form biofilms for protection. Therefore, the bacteria could be more susceptible to antimicrobial treatment. Like it is mentioned for *Bacillus subtilis*, glucose first enhances and afterwards inhibits the transcription of *ica*ADBC [30]. 

The PIA protein, which is encoded by the *ica*ADBC locus, mediates the biofilm accumulation and formation of *S. epidermidis* [39,40]. Additionally, it is also involved on the bacterial adhesion mechanisms [41]. PIA production is linked to the expression of all four genes *ica*A, *ica*D, *ica*C and *ica*B. Furthermore, the synthesis of PIA is dependent on environmental influences like anaerobic states, salt, glucose, alcohol concentration and antibiotics [42]. Several regulators are associated with the PIA production. If the most prominent regulator, icaR, is deleted, the PIA production is upregulated [43]. Furthermore, aminoglycoside antibiotics, like gentamicin, can have an influence on the binding of icaR to the DNA and so induce biofilm formation [44]. In our study, the results for gene expression resulted in a very low relative gene expression. This could be due to the existence of ica-independent biofilm mechanisms like by the production of the accumulation-associated protein (Aap) or the major cell wall autolysin *atl*E and accumulation of extracellular DNA [45].

All these findings encouraged us in our hypothesis, that the use of different nutrient media not only alters the growth, viability and gene expression of CoNS, but also alters the susceptibility to different antibiotics when compared to human SF. Furthermore, the limitations involved in the obtainment of sterile SF from patients leads to the urgent need of more research on a medium which is reproducible, allowing high throughput and at the same time simulates the tissue fluids and implant surroundings. 

In our study we only used patient isolated strains. Patient isolated strains usually grow slowly in vitro and the use of nutrient-rich media could help dealing with such difficulties. The cultivation of these strains in human SF is a limiting factor. Therefore, a use of standard laboratory strains would result in faster results and less waste of materials needed for repetitions. However, it would still be far from reality of natural biofilm environments as for example in PJI patients. In addition, the combination of simulated body fluid and tissue engineering could help to reduce animal testing and at the same time, offer more accurate and realistic results for the treatment of PJIs. Such steps could improve the transferability of in vitro test results to the clinical reality. An alternative would be the use of artificially simulated fluids. The use of simulated fluids like artificial sputum, simulated gastric fluid or other tissular fluids like lung fluid or simulated semen fluid are already used to enhance the in vitro study of various diseases and reduce the demand on animal experiments [46]. Simulated SF should be easy to produce, more ethical and should offer high throughput in the laboratorial routine. This simulated SF could then be adapted for certain human joint diseases like osteoarthritis and rheumatoid arthritis [47,48]. Further studies on the production of simulated body fluids and standardization of media for research and clinical tests for biofilm-related infections should be encouraged. 

For observation of the biofilm cell viability, we used an XTT reduction assay. XTT is a tetrazolium salt with no color. It is converted into a water-soluble formazan derivate by dehydrogenases of metabolic active bacteria [49]. A limitation of this method for the evaluation of biofilms is that cell viability measurements are just a snapshot in time. Biofilms also tend to consist of metabolic active and metabolic inactive or dormant bacteria [50]. These bacteria usually have a higher resistance to antibiotics and cannot be detected by a cell viability test which concentrates on metabolism. 

## 5. Conclusions

The results highlight the influence of glucose on the antibiotic susceptibility, growth and gene expression of all strains tested. For all strains, a significant difference in bacterial recovery, viability and gene expression were found when compared to biofilm grown in SF. For all bacteria, the use of SF as nutrient medium, showed significant differences to other media when antibiotic susceptibility, growth and *ica*ADBC gene expression were investigated. This leads us to the conclusion that nutrient media used in laboratories to detect antibiotic susceptibility have a high influence on its results, therefore, the use of media which are more patient fluid-like should be considered.

## Figures and Tables

**Table 1:** Forward and reverse primers for all strains used in this study.

Genes	Strains	Primer Sequences	Amplicon Length (bp)	References
16S rRNA	All strains	**F:** 5′-GAAAGCCACGGCTAACTACG-3′ **R:** 5′-CATTTCACCGCTACACATGG-3′	368	[23]
*ica*A	*S. epidermidis*	**F:** 5′-TCTCTTGCAGGAGCAATCAA-3′ **R:** 5′-TCAGGCACTAACATCCAGCA-3′	188	[24]
	*S. simulans*	**F:** 5′-GGAGGTCTTTGGAAGCAACG-3′ **R:** 5′-AACCTTTTCGTTTTCATTGTGCT-3′	92	This study
	*S. lugdunensis*	**F:** 5′- ACCTTGTGCCCATCGAAGAC-3′ **R:** 5′-TGCGGTAACTGGCAATCCTC-3′	337	This study
	*S. haemolyticus*	**F:** 5′-TTTGCTGGATGTTGGTGCCT-3′ **R:** 5′-ACTTCATGCCCACCTTGAGC -3′	79	This study
*ica*D	*S. epidermidis*	**F:** 5′-ATGGTCAAGCCCAGACAGAG-3′ **R:** 5′-CGTGTTTTCAACATTTAATGCAA-3′	198	[24]
	*S. simulans*	**F:** 5′- ACTCATGTGGTACAGCTCTCTT-3′ **R:** 5′-GACAAGTCCAGACAGAGGGA-3′	307	This study
	*S. lugdunensis*	**F:** 5′- TGTCACTCATCGTAACTGCTTC-3′ **R:** 5′- ATCCATGCTTTTTGCCATGC-3′	178	This study
	*S. haemolyticus*	**F:** 5′- AAGCCCAGACAGAGGCAATA -3′ **R:** 5′-CAAACAAACTCATCCATCCGAA -3′	229	This study
*ica*B	*S. epidermidis*	**F:** 5′-AATGACGAATGCAACACCAA-3′ **R:** 5′-CGTGTGCTTTAAGCCATTGA-3′	227	[25]
	*S. simulans*	**F:** 5′-GGTCCCCTACATGATCCGTT-3′ **R:** 5′-ACTTGGTGGCGAGAAAAATGG-3′	350	This study
	*S. lugdunensis*	**F:** 5′- TCCAGACACTTTTAGGTGGGA-3′ **R:** 5′- CTTAAAGCACATGGGGCACA-3′	88	This study
	*S. haemolyticus*	**F:** 5′- AGCGCACTCGCGTTAAACTA -3′ **R:** 5′-AACTTTGCGTCGTGTGCTTT -3′	158	This study
*ica*C	*S. epidermidis*	**F:** 5′-CGCTGTTTCCGGTAGTGATT-3′ **R:** 5′-TTGGGTGCAACAAATAAATGA-3′	176	[26]
	*S. simulans*	**F:** 5′- TCCGCATATCACAGAGTTCCA-3′ **R:** 5′- AAGCACGGTGTCGCACTAAA-3′	75	This study
	*S. lugdunensis*	**F:** 5′- TAAAGCAAGGCGTGCCAAAG-3′ **R:** 5′- ATGCGAGGTATTGATGGCGA-3′	88	This study
	*S. haemolyticus*	**F:** 5′- TCGCTGTTTCCGGTAGTGATT -3′ **R:** 5′-TCCAGTTAGGCTGGTATTGGTC -3′	372	This study

**Table 2:** Accession numbers for the genes and their corresponding strains used.

Strains	Genes	Accession Numbers
*S. simulans*	*ica*A	AF500263.1
	*ica*D	NZ_KQ957518.1
	*ica*B	NZ_KQ957518.1
	*ica*C	LR134264.1
*S. lugdunensis*	*ica*A	FR870271.1
	*ica*D	FR870271.1
	*ica*B	FR870271.1
	*ica*C	FR870271.1
*S. haemolyticus*	*ica*A	FJ472951
	*ica*D	FJ472951
	*ica*B	FJ472951
	*ica*C	FJ472951

**Table 3:** Comparison of MIC and BIC testing for five different coagulase-negative *staphylococci* strains.

**MIC (µg/mL)**					
**Antibiotics**	***S. epidermids* R**	***S. epidermids***	***S. simulans***	***S. lugdunensis***	***S. haemolyticus***
Gentamicin	3	0.094	0.016	0.016	4
Clindamycin	>256	0.064	0.016	0.023	2
Vancomycin	1	1.5	0.5	1.5	1.5
Rifampicin	>32	0.012	0.003	0.008	0.008
**BIC (µg/mL)**					
**Antibiotics**	***S. epidermids* R**	***S. epidermids***	***S. simulans***	***S. lugdunensis***	***S. haemolyticus***
Gentamicin	>1024	256	512	>1024	>1024
Clindamycin	>1024	>1024	>1024	>1024	>1024
Vancomycin	>1024	>1024	>1024	>1024	>1024
Rifampicin	>1024	>1024	>1024	>1024	1

**Table 4:** Concentration of the antibiotics used in this study.

Antibiotic Concentrations (µg/mL)					
Antibiotics	*S. epidermids* R	*S. epidermids*	*S. simulans*	*S. lugdunensis*	*S. haemolyticus*
Gentamicin	1024	16	16	64	256
Clindamycin	1024	16	16	1024	256
Vancomycin	32	16	64	1024	1
Rifampicin	8	4	0.25	0.25	0.5

## Data Availability

Data is contained within the article.

## Supplementary Materials

The following are available online at https://www.mdpi.com/article/10.3390/antibiotics10070790/s1, Table S1: List of *p*-values for biofilm cell recovery after antibiotic treatment; Table S2: List of *p*-values for biofilm cell viability after antibiotic treatment; Table S3: List of *p*-values for biofilm growth; Table S4: List of *p*-values for biofilm viability; Table S5: List of *p*-values for biofilm gene expression of icaADBC.
