# Supplementary material for: Influence of Nutrient Media Compared to Human Synovial Fluid on the Antibiotic Susceptibility and Biofilm Gene Expression of Coagulase-Negative Staphylococci In Vitro
Source: Antibiotics (Basel). 2021 Jun 29;10(7):790. doi: 10.3390/antibiotics10070790 (PMC8300679; doi:10.3390/antibiotics10070790)
Supplement: Supplementary file 1 [file antibiotics-10-00790-s001.zip › antibiotics-1272388-supplementaryV4.pdf]

Table S1. List of *p*-values for biofilm cell recovery after antibiotic treatment.

P-Values Compared to SF						
Nutrient medium	Antibiotic treatment	*S. epidermids* R	*S. epidermids*	*S. simulans*	*S. lugdunensis*	*S. haemolyticus*
TSB+G	gentamicin	0.0451	0.0213	ns	<0.0001	ns
	clindamycin	0.0355	ns	<0.0001	<0.0001	<0.0001
	vancomycin	ns	<0.0001	ns	0.0014	<0.0001
	rifampicin	ns	ns	ns	ns	ns
TSB	gentamicin	0.0036	ns	0.0011	<0.0001	0.0339
	clindamycin	0.0004	ns	<0.0001	<0.0001	0.0001
	vancomycin	ns	<0.0001	ns	0.0114	<0.0001
	rifampicin	0.0315	ns	ns	ns	0.0045
MHB+G	gentamicin	ns	0.0362	ns	<0.0001	ns
	clindamycin	0.0317	ns	<0.0001	<0.0001	<0.0001
	vancomycin	ns	<0.0001	ns	0.0004	<0.0001
	rifampicin	0.0017	ns	ns	ns	ns
MHB	gentamicin	ns	ns	0.0386	<0.0001	ns
	clindamycin	ns	ns	<0.0001	<0.0001	<0.0001
	vancomycin	0.0053	<0.0001	ns	<0.0001	<0.0001
	rifampicin	0.0255	ns	ns	ns	ns

P-Values between +/- 1% Glucose						
Nutrient medium	Antibiotic treatmen	*S. epidermids* R	*S. epidermids*	*S. simulans*	*S. lugdunensis*	*S. haemolyticus*
TSB+G TSB	gentamicin	ns	ns	0.0390	ns	ns
	clindamycin	ns	ns	ns	ns	ns
	vancomycin	ns	ns	ns	ns	ns
	rifampicin	0.0315	ns	ns	ns	ns
MHB+GM HB	gentamicin	ns	ns	ns	ns	ns
	clindamycin	ns	ns	ns	ns	ns
	vancomycin	ns	<0.0001	ns	ns	ns
	rifampicin	ns	ns	ns	ns	ns
SF+G SF	gentamicin	ns	<0.0001	ns	<0.0001	ns
	clindamycin	<0.0001	ns	0.0343	ns	ns
	vancomycin	0.0007	<0.0001	0.0099	ns	ns
	rifampicin	0.0002	<0.0001	ns	ns	ns

ns = no significance.

Table S2. List of *p*-values for biofilm cell viability after antibiotic treatment.

P-Values Compared to SF						
Nutrient medium	Antibiotic treatment	*S. epidermids* R	*S. epidermids*	*S. simulans*	*S. lugdunensis*	*S. haemolyticus*
TSB+G	gentamicin	<0.0001	<0.0001	ns	<0.0001	ns
	clindamycin	<0.0001	ns	0.0003	0.0001	ns
	vancomycin	ns	<0.0001	ns	<0.0001	<0.0001
	rifampicin	0.0008	0.0299	ns	ns	ns
TSB	gentamicin	0.0005	<0.0001	ns	<0.0001	ns
	clindamycin	<0.0001	0.0465	0.0007	<0.0001	0.0180
	vancomycin	0.0229	<0.0001	ns	<0.0001	<0.0001

	rifampicin	<0.0001	0.02228	ns	ns	ns
MHB+G	gentamicin	<0.0001	<0.0001	ns	<0.0001	ns
	clindamycin	<0.0001	ns	0.0083	<0.0001	0.0313
	vancomycin	ns	<0.0001	ns	<0.0001	<0.0001
	rifampicin	0.0006	0.0285	ns	ns	ns
MHB	gentamicin	<0.0001	<0.0001	ns	<0.0001	ns
	clindamycin	<0.0001	ns	ns	<0.0001	<0.0001
	vancomycin	<0.0001	ns	ns	<0.0001	<0.0001
	rifampicin	<0.0001	0.0292	ns	ns	ns

P-Values between +/- 1% Glucose						
Nutrient medium	Antibiotic treatmen	*S. epidermids* R	*S. epidermids*	*S. simulans*	*S. lugdunensis*	*S. haemolyticus*
TSB+G TSB	gentamicin	0.0058	0.0399	ns	ns	ns
	clindamycin	ns	ns	ns	ns	ns
	vancomycin	ns	ns	ns	ns	ns
	rifampicin	<0.0001	ns	ns	ns	ns
MHB+GMHB	gentamicin	ns	ns	ns	ns	ns
	clindamycin	<0.0001	ns	ns	ns	0.0033
	vancomycin	<0.0001	0.0420	ns	ns	ns
	rifampicin	ns	ns	ns	ns	ns
SF+G SF	gentamicin	ns	ns	ns	0.0373	ns
	clindamycin	ns	0.0032	ns	ns	0.0033
	vancomycin	<0.0001	ns	ns	0.0116	ns
	rifampicin	ns	ns	ns	<0.0001	ns

ns = no significance.

Table S3 List of *p*-values for biofilm growth.

P-Values Compared to SF					
Nutrient medium	*S. epidermids* R	*S. epidermids*	*S. simulans*	*S. lugdunensis*	*S. haemolyticus*
TSB+G	ns	ns	<0.0001	ns	<0.0001
TSB	ns	ns	<0.0001	ns	<0.0001
MHB+G	ns	ns	<0.0001	ns	<0.0001
MHB	0.0008	ns	<0.0001	ns	<0.0001
**P-Values between +/- 1% Glucose**					
Nutrient medium	*S. epidermids* R	*S. epidermids*	*S. simulans*	*S. lugdunensis*	*S. haemolyticus*
TSB+G TSB	ns	ns	ns	ns	ns
MHB+GMHB	ns	ns	ns	ns	ns
SF+G SF	0.0111	<0.0001	ns	ns	0.0240

ns = no significance.

Table S4 List of *p*-values for biofilm viability.

**P-Values Compared to SF**					
Nutrient medium	*S. epidermids* R	*S. epidermids*	*S. simulans*	*S. lugdunensis*	*S. haemolyticus*
TSB+G	0.0001	<0.0001	<0.0001	<0.0001	ns
TSB	<0.0001	<0.0001	<0.0001	<0.0001	<0.0001
MHB+G	ns	ns	ns	0.0017	ns
MHB	<0.0001	0.0007	0.0024	ns	<0.0001
**P-Values between +/- 1% Glucose**					
Nutrient medium	*S. epidermids* R	*S. epidermids*	*S. simulans*	*S. lugdunensis*	*S. haemolyticus*
TSB+G TSB	<0.0001	<0.0001	0.0031	ns	<0.0001
MHB+GMHB	<0.0001	ns	<0.0001	0.0002	<0.0001
SF+G SF	ns	ns	<0.0001	ns	<0.0001

ns = no significance.

Table S5 List of *p*-values for biofilm gene expression of *ica*ADBC.

**P-Values Compared to SF**						
Nutrient medium	Antibiotic treatment	*S. epidermids* R	*S. epidermids*	*S. simulans*	*S. lugdunensis*	*S. haemolyticus*
TSB+G	*ica*A	ns	<0.0001	<0.0001	<0.0001	<0.0001
	*ica*D	ns	ns	0.0407	<0.0001	<0.0001
	*ica*B	ns	ns	ns	<0.0001	ns
	*ica*C	ns	<0.0001	<0.0001	<0.0001	<0.0001
TSB	*ica*A	ns	<0.0001	<0.0001	<0.0001	<0.0001
	*ica*D	0.0003	ns	0.0368	<0.0001	<0.0001
	*ica*B	0.0041	ns	<0.0001	<0.0001	<0.0001
	*ica*C	0.0094	<0.0001	ns	<0.0001	<0.0001
MHB+G	*ica*A	<0.0001	ns	<0.0001	<0.0001	ns
	*ica*D	<0.0001	ns	0.0368	<0.0001	ns
	*ica*B	<0.0001	0.0001	<0.0001	<0.0001	<0.0001
	*ica*C	<0.0001	<0.0001	<0.0001	<0.0001	ns
MHB	*ica*A	<0.0001	<0.0001	<0.0001	ns	<0.0001
	*ica*D	<0.0001	ns	0.0368	0.0415	ns
	*ica*B	<0.0001	<0.0001	<0.0001	ns	<0.0001
	*ica*C	<0.0001	<0.0001	ns	ns	ns

**P-Values between +/- 1% Glucose**						
Nutrient medium	Antibiotic treatmen	*S. epidermids* R	*S. epidermids*	*S. simulans*	*S. lugdunensis*	*S. haemolyticus*
TSB+G TSB	*ica*A	ns	ns	ns	<0.0001	<0.0001
	*ica*D	ns	ns	<0.0001	<0.0001	<0.0001
	*ica*B	ns	ns	<0.0001	<0.0001	<0.0001
	*ica*C	ns	<0.0001	<0.0001	<0.0001	<0.0001
MHB+GMHB	*ica*A	<0.0001	<0.0001	ns	<0.0001	<0.0001
	*ica*D	<0.0001	ns	ns	<0.0001	ns
	*ica*B	<0.0001	<0.0001	<0.0001	<0.0001	ns

	*ica*C	<0.0001	ns	<0.0001	<0.0001	ns
	*ica*A	ns	<0.0001	<0.0001	ns	ns
SF+G	*ica*D	ns	ns	ns	ns	<0.0001
SF	*ica*B	ns	ns	0.0070	ns	<0.0001
	*ica*C	ns	<0.0001	ns	ns	ns

ns = no significance.
